# Upper Gastro-Intestinal Endoscopy in a General Surgical Unit

**Published:** 1986-08

**Authors:** Colin D. Johnson

**Affiliations:** Department of Surgery, Southmead Hospital, Bristol

## Abstract

A consecutive series of 106 upper gastrointestinal endoscopies on a general surgical firm is reviewed. Early endoscopy provided a diagnosis in 86% of patients with Gl bleeds. In patients with a firm radiological diagnosis of duodenal ulcer or gastro-oesophageal reflux, endoscopy added little to management, but endocscopy was helpful when radiology was equivocal, for gastric lesions and for oesophageal strictures. Therapeutic endoscopy was helpful in management on nine occasions.


### Upper gastrointestinal endoscopy in a general surgical unit

**Colin D. Johnson, FRCS**
Department of Surgery, Southmead Hospital, Bristol

### SUMMARY

A consecutive series of 106 upper gastrointestinal endoscopies on a general surgical firm is reviewed. Early endoscopy provided a diagnosis in 86% of patients with GI bleeds. In patients with a firm radiological diagnosis of duodenal ulcer or gastro-oesophageal reflux, endoscopy added littlé to management, but endocscopy was helpful when radiology was equivocal, for gastric lesions and for oesophageal strictures. Therapeutic endoscopy was helpful in management on nine occasions.

### INTRODUCTION

In view of the continuing debate over the relative merits of double-contrast radiology and gastroscopy (1, 2), a survey was made of the current status of gastroscopy on a general surgical firm, in an attempt to define its role.

### METHODS

During a 5-month period, the author performed all the gastroscopies on the firm (2 consultants, senior registrar and house staff), which covers the full range of general surgery. Another consultant surgeon provides the ERCP service in the hospital, in addition to gastroscopy and colonoscopy.

The study was prospective. The history and findings were recorded, and a note was made whether a diagnosis had been achieved, confirmed, or was still in doubt. If a biopsy was taken, the histological findings were classified as confirmatory of the endoscopic diagnosis, or diagnostic if that had been in doubt, or was disproved by the histology.

The effect of gastroscopy on management was recorded as either confirmation or change in the planned medical or surgical treatment. Patients treated for the first time after a diagnostic endoscopy were classed as changes in management.

The case notes were reviewed 2–6 months after endoscopy to obtain a final diagnosis.

### RESULTS

One hundred and six endoscopies were performed in 98 patients, mean age 61 years (range 15–90).

### *Epigastric pain*

This was the main indication for 54 endoscopies, alone in 48 and with other major symptoms in six. Seventeen patients had a final diagnosis of duodenal ulcer or duodenitis. Endoscopy contributed to diagnosis in 12 patients, but in 8 of these served only to confirm radiological findings. Endoscopy was part of preoperative assessment in 5 patientts (4 operated, 1 avoided operation). One wrong diagnosis was made. A 74-year-old man presented with epigastric pain and vomiting. Barium meal showed a 'probable carcinoma of the pylorus', and gastroscopy confirmed this, but the biopsy report was 'benign ulceration and inflammation with dysplasia'. A second gastroscopy after three weeks treatment showed a 'pyloric channel ulcer, ?malignant'. The biopsy was reported as showing a moderatly differentiated adenocarcinoma. He underwent partial gastrectomy, but examination of the specimen showed duodenal ulceration with no evidence of malignancy.

Seven patients had a hiatus hernia with reflux oesophagitis. Medical management was confirmed in four, one of whom required surgery three months later. Medical anti-reflux measures were started in two patients after diagnostic gastroscopy. The seventh patient had gallstones and was offered fundoplication at the time of cholecystectomy.

Eight endoscopies were done in 7 patients thought to have gastric ulcers. In 6 the diagnostis was confirmed, but one was shown to have a carcinoma, confirmed by gastrectomy.

Four patients had gastritis with bile reflux. Gastroscopy was required to make the diagnosis in three, and was done in the fourth to assess response to treatment.

Twelve examinations were normal, supporting an extra-gastric diagnosis in 5 (irritable colon 1, scar tenderness 1, depression 3). In 9 of these patients existing management was confirmed.

### *Patients without epigastric pain*

Four patients had dysphagia. One peptic stricture of the oesophagus was confirmed, but one radiologically benign stricture appeared malignant at endoscopy, a finding confirmed by histology, with a resulting change to surgical management. Two other carcinomas of the oesophagus were confirmed by endoscopy.

Two patients had barium meals suggesting benign pyloric stenosis. Gastroscopy showed both to have carcinomas. Endoscopy was done in one patient each to confirm a clinical diagnosis of gastric cancer, extra-gastric mass and globus hystericus. One patient with polyposis coli was examined to exclude gastroduodenal polyps.

### *Gastrointestinal bleeding*

Twenty eight patients presented with haematemesis or melaena and two with anaemia. Ten patients had peptic ulcers (DU 5; GU 4, stomal 1) and one had a carcinoma of the pylorus.

Eleven patients had hiatus hernias. There was oesophagitis in 10, with ulceration in 4. One patient had a Mallory-Weiss tear. Additional anti-reflux therapy was given to six of these patients following endoscopy.

Leiomyoma of the stomach, gastritis and oesophageal varices were found in one patient each. In 4 patients (13%) no source of bleeding was found.

### *Therapeutic endoscopy*

Seven oesophageal dilatations were performed in six patients, including two undergoing repair of a hiatus hernia. One stricture proved to be malignant and was intubated endoscopically. A further patient underwent endoscopic removal of a gastric polyp.

*Barium meal and endoscopy*

Four patients had had a barium meal more than two years before endoscopy. A new diagnosis wsa made in each case.

Of 30 patients in whom endoscopy followed barium meal within a few weeks or months, gastroscopy was helpful only in oesophageal strictures and gastric lesions. In 12 patients with duodenal ulcer or oesophageal reflux there was no change in management as a result of the endoscopy, although 2 unsuspected duodenal ulcers were found in patients with hiatus hernias.

Endoscopy played a useful role in the management of all patients with oesophageal obstruction, resulting in a change in the surgical plan in three. Two others had histological confirmation of cancer before surgery and one doubtful stricture was shown to be due to oesophagitis.

Surgical management of two patients with carcinoma of the pylorus was changed following endoscopy, when prior barium study had suggested benign pyloric stenosis. Five patients had gastroscopy to confirm a radiological diagnosis of gastric ulcer; one of these was found to have a carcinoma.

### DISCUSSION

While controversy continues over whether endoscopy or barium meal should be the initial investigation (1, 2), it is recommended that both examinations need be performed only in selected patients (3, 4). In this district, general practitioners may request barium meals, but not endoscopy. Patients with radiological abnormalities are referred for further investigation at the discretion of the surgeon. In this study, endoscopy did not alter the management of patients with radiologically demonstrated duodenal ulceration or oesophageal reflux, although two out of six patients with hiatus hernia were found to have duodenal ulcers as well.

This lack of therapeutic benefit of endoscopy in some categories of patient must be seen in the context of the time spent performing endoscopy. Half a day case list each week is allocated to endoscopy; about half the cases were done in this time. The remainder occupied the equivalent of one operating list a month. About half the patients investigated for epigastric pain continued on the same treatment after endoscopy. A pre-existing diagnosis was confirmed in 35 of 68 endoscopies.

In contrast, radiology was unreliable in the diagnosis of pyloric stenosis, and endoscopy was most useful in the diagnosis and management of oesophageal strictures. It could be argued that after a barium meal endoscopy is only indicated for obstructing lesions of the oesophagus and pylorus, to obtain histology of gastric ulcers and in the elucidation of equivocal radiological findings. In two patients treatment was delayed while awaiting endoscopy after radiological diagnosis (linitis plastica and Crohn's disease of the duodenum).

In patients with typical symptoms and radiological reflux, endoscopy always showed oesophagitis. Fraser and Earnshaw (4) also found a good correlation between radiology and endoscopy in this condition.

Lockhart *et al* (5) studied 100 elderly patients having endoscopy, of whom 46 had both endoscopy and a barium meal. In just over half of these examinations the radiological diagnosis was confirmed, which is in agreement with the present series. They too found that endoscopy after barium meal is of most value for gastric lesions and oesophageal obstruction.

The high rate of diagnosis in patients presenting with gastrointestinal bleeding (86%) compares well with that reported by others (6), and was probably achieved as a result of our policy of endoscopy within 24 hours of referral by the admitting physician. Endoscopy was useful in this group of patients. Thirty-six per cent were bleeding from oesophageal ulcers or oesophagitis, and early diagnosis allowed the addition of antireflux measures to existing antisecretory therapy. Patients with peptic ulcers underwent surgery on the basis of clinical criteria. In fact, all those who required surgery had a large vessel visible in the base of the ulcer.

In this series, therapeutic endoscopy was usually for the relief of dysphagia, by dilatation or intubation. One patient was spared a laparotomy by endoscopic polypectomy, but gastric polyps are rare. Therapeutic endoscopy always benefitted the patients, with no complications. The usefulness of therapeutic endoscopy is strong justification for the use of operating time to provide a within-firm endoscopy service.

In conclusion, there was greatest benefit from therapeutic endoscopy and in the diagnosis of gastrointestinal haemorrhage. In the investigation of upper gastrointestinal symptoms gastroscopy was useful if no barium meal had been done in the preceding two years, and in the assessment of radiologically demonstrated gastric ulcers and oesophageal or pyloric obstruction. There was no benefit from endoscopy after a barium meal showing duodenal ulcer or oesophageal reflux.

### REFERENCES

1. DOOLEY, C. P., LARSON, A. W., STACE., N. H. *et al.* (1984) Double contrast barium meal and upper gastrointestinal endoscopy. *Ann.Int.Med.,* **101**, 538–545.
2. OUYANG, A. (1985) Radiology *vs* endoscopy. *Gastroenterol,* **88**, 1281–1283.
3. SIVAK, M. V. (1984) Upper gastrointestinal endoscopy. When to perform, what to expect. *Postgrad.Med.* **75**, 81–87.
4. FRASER, G. M., EARNSHAW, P. M. (1983) The double contrast barium meal: a correlation with endoscopy. *Clin.Radiol.* **34**, 121–131.
5. LOCKHART, S., SCHOFIELD, P. M., GRIBBLE, R. J. N., BARON, J. H. (1985) Upper gastrointestinal endoscopy in the elderly. *Br.Med.J.* **290**, 283.
6. DRONFIELD, M. W., LANGMAN, M. J. S., ATKINSON, M. *et al.* (1983) Outcome of endoscopy and barium radiography for acute upper gastrointestinal bleeding: controlled trial in 1037 patients. *Br.Med.J.* **284**, 545–548.

*Address for Correspondence:* Department of Surgery, St Stephen's Hospital, Fulham Road, London.

